# Linking hypoxia, DNA damage and proliferation in multicellular tumor spheroids

**DOI:** 10.1186/s12885-017-3319-0

**Published:** 2017-05-18

**Authors:** Stephen Riffle, Ram Naresh Pandey, Morgan Albert, Rashmi S. Hegde

**Affiliations:** Division of Developmental Biology, Cincinnati Children’s Hospital Medical Center, University of Cincinnati College of Medicine, 3333 Burnet Avenue, Cincinnati, OH 45229 USA

**Keywords:** Spheroid, Ewing sarcoma, DNA damage repair, ATM, ATR, Hypoxia, Proliferation

## Abstract

**Background:**

Multicellular Tumor Spheroids are frequently used to mimic the regionalization of proliferation and the hypoxic environment within avascular tumors. Here we exploit these features to study the activation of DNA damage repair pathways and their correlation to developing hypoxia.

**Methods:**

Activation of DNA damage repair markers, proliferation, cell death, glycogen accumulation and developing hypoxia were investigated using immunofluorescence, immuno-histochemistry, EdU incorporation, Western blots, COMET assays, and pharmacological agents in A673 Ewing sarcoma spheroids and monolayer cultures.

**Results:**

DNA damage marker γ-H2AX is observed in the hypoxic, peri-necrotic region of growing spheroids. While most proliferating cells are seen on the spheroid surface, there are also a few Ki-67 positive cells in the hypoxic zone. The hypoxia-induced phosphorylation of H2AX to form γ-H2AX in spheroids is attenuated by the ATM inhibitor KU55933, but not the ATR inhibitor VE-821.

**Conclusion:**

Tumor spheroids mimic tumor microenvironments such as the anoxic, hypoxic and oxic niches within solid tumors, as well as populations of cells that are viable, proliferating, and undergoing DNA damage repair processes under these different micro-environmental conditions. ATM, but not ATR, is the primary kinase responsible for γ-H2AX formation in the hypoxic core of A673 spheroids. Spheroids could offer unique advantages in testing therapeutics designed to target malignant cells that evade conventional treatment strategies by adapting to the hypoxic tumor microenvironment.

## Background

The growth of solid tumors is accompanied by the development of central hypoxic or anoxic regions, an acidic extracellular pH and modified cellular glucose and energy metabolism. Tumor cells either adapt to the unfavorable microenvironment or die, leading to areas of necrosis. Continued growth of the tumor then becomes dependent on the delivery of oxygen and nutrients through vascularization. A primary response of tumor cells to hypoxia is to upregulate HIF1-α and its downstream targets, principally the pro-angiogenic VEGF. However, because of its leaky and inefficient nature, tumor angiogenesis does not completely counter tumor hypoxia which can range from near anoxia (0.02% oxygen tension) at distances over 150 μm from vessels, to moderate (8% oxygen tension) hypoxia [1]. Clinically relevant consequences of tumor hypoxia include increased chemo- and radiation-resistance, increased metastatic potential, genomic instability and poorer prognosis.

Another defining feature of vascularized tumors is the dynamic variation in localized oxygen tension as the unstable tumor vasculature transiently provides re-oxygenation. A consequence of these cycles of ischemia and reperfusion is the localized formation of reactive oxygen species (ROS) [2]. The effect of hypoxia and ROS on cell survival, replication and DNA damage has been extensively studied in both tumor and non-tumor cells (reviewed in [3–7]). Severe hypoxia (<0.2% O2) can cause S-phase arrest, ATR activation and phosphorylation of H2AX-Ser139 (γ-H2AX), p53-Ser15, Chk1-Ser345, and Chk2-Thr68. Moderate hypoxia can lead to replication stress and activation of the DNA Damage Repair (DDR) pathway proteins even in the absence of measurable DNA damage [8–10]. In contrast, hypoxia-reoxygenation induces DNA damage through ROS and leads to ATM-dependent H2AX and p53 phosphorylation [2]. The cellular response to DNA damage is to limit proliferation while initiating repair. The coordinated regulation of the ATM/ATR pathways is critical to this process.

Extensive studies associating DNA damage, the upregulation of DNA damage repair pathways, and hypoxia have been conducted on a variety of cell types in 2D–cell culture systems. However, a better representation of the tumor microenvironment is provided by 3D culture models such as multi-cellular tumor spheroids (MCTS). In such spheroids oxygenation status is determined by the rates of oxygen diffusion and consumption alone, without the complicating influence of the tumor vasculature. MCTS are also a preferred platform for testing in-vitro drug sensitivity which can be either potentiated or suppressed by MCTS-specific features such as the 3D microenvironment (hypoxia, acidosis) and cell aggregation [11]. These features of MCTS lead us to the hypothesis that MCTS could be used to study the relationship between clinically relevant features of a hypoxic microenvironment such as proliferation and activation of DNA damage repair pathways. To date, there is no good model available for the study of hypoxia induced changes within spatially defined cell populations. The lack of such a model limits our ability to study relevant mechanisms of cell survival and to accurately predict efficacy of targeted therapeutics in the unique hypoxic environment present only in 3D and in vivo settings. To recapitulate features of the hypoxia-induced cellular response in avascular tumors (or at sites distant from blood vessels), here we use MCTS to study the spatial correlation between the development of hypoxia, cell proliferation, cell death, and the activation of DDR components. We show that developing hypoxia in the core of growing MCTS is accompanied by a distinctive distribution of proliferating cells, glycogen accumulation, HIF1-α, activated ATM kinase, and induction of γ-H2AX. Using pharmacological inhibitors of ATM and ATR we distinguish between their contributions to H2AX phosphorylation and cell proliferation in the differing microenvironments represented within an MCTS. Our results demonstrate that MCTS can be a physiologically relevant and convenient system for studying the activation of DNA damage-associated pathways in heterogeneous tumor cell populations present within solid tumors, and their susceptibility to pharmacological intervention.

## Methods

### Cell lines, antibodies and reagents

Human Ewing Sarcoma A673 (CRL-1598; ATCC, Manassas, VA) and LLC (CRL-1642; ATCC, Manassas, VA); Dulbecco’s Modified Eagles Medium, DMEM, (11,965,092; Thermoscientific, Waltham, MA); Fetal Bovine serum - FBS, (TMS-013-B; Millipore, Billerica, MA); Agar (J637; Amresco, Solon, OH); DMSO (67–68-5, Sigma, St. Louis, MO); Proox-p110 ProCO_2_ Hypoxia Chamber (Biospherix, Parish, NY); Inflatable Glove Bag Model X-37-27 (108D; Glas-Col, Terre Haute, IN); ATM inhibitor KU55933 (S1092; Selleckchem, Houston, TX); ATR inhibitor VE-821 (S8007; Selleckchem, Houston, TX); Click-IT Plus EdU imaging kit (MP10637, Life technologies, Carlsbad, CA).

The following antibodies were used in these studies: mouse anti-γ-H2AX (JBW301; Millipore, Billerica, MA), rabbit anti-Ki-67 (MA5–1452; Thermoscientific, Waltham, MA), rabbit anti-cleaved caspase-3 (5A1E; Cell Signaling Technology, Danvers, MA), mouse anti-HIF1-α (810,958; BD Biosciences, San Jose, CA), mouse anti-phospho-serine 1981 ATM (05–740; Millipore, Billerica, MA), rabbit anti-phospho-serine 428 ATR (2853P; Cell Signaling Technology, Danvers, MA), mouse anti-pimonidazole (HP1; Hypoxyprobe, Burlington, MA), goat anti-mouse IgG (H + L) Alexa Fluor 647 (A-21235; Thermoscientific, Waltham, MA), goat anti-mouse IgG Cyanine 5 (A-10524; Thermoscientific, Waltham, MA), donkey anti-rabbit Alexa Fluor 594 (A-21027; Thermoscientific, Waltham, MA), rabbit anti-phosphoserine 345 Chk1 (2341; Cell Signaling Technology, Danvers, MA), horse radish peroxidase-conjugated goat anti-mouse (A120-11P; Bethyl, Montgomery, TX), horse radish peroxidase-conjugated goat anti-mouse (A120-201P; Bethyl, Montgomery, TX).

### Cell culture

A673 and LLC were maintained in standard culture conditions (37 °C with 5% CO_2_) in DMEM with 1% vv^−1^ penicillin (100 IU ml^−1^) and streptomycin 100 mg ml^−1^ supplemented with 10% vv^−1^ heat inactivated FBS.

### MCTS culture

Spheroids were formed through use of the liquid overlay method wherein single cell suspensions (100 μl of A673 or LLC cell suspensions in DMEM supplemented with 2% FBS or 10% FBS were added at a density of 5 × 10^4^ or 5 × 10^3^ cells ml^−1^ respectively) were added to 96-well plates previously coated with agar. The agar coating prevents cell adhesion. Plates containing single cell suspensions were incubated stationary in standard cell culture conditions (37 °C, 5% CO_2_) for 48 h. During this time, tumor cells aggregated forming a single, connected mass of cells that could be moved as a singular unit. After this 48-h formation period, 100 μl of fresh media was added to each well. This makes for a total of 200 μl of total media in each well. After this point, 100 μl of media was removed and replaced with fresh media every 48 h such that total volume within the wells was maintained at 200 μl. In experiments with test compounds or hypoxia treatment, spheroids were individually washed with DMEM and transferred to freshly coated agar plates with 200 μl of fresh medium containing test inhibitors when applicable.

### Hypoxia treatment

Hypoxia treatment was performed at 37 °C for 12 h with 1% O_2_, 5% CO_2_ in a hypoxia chamber followed by collection in a hypoxic glove bag pre-equilibrated at 1% O_2_.

### MCTS growth curves

**Fig. 1:**
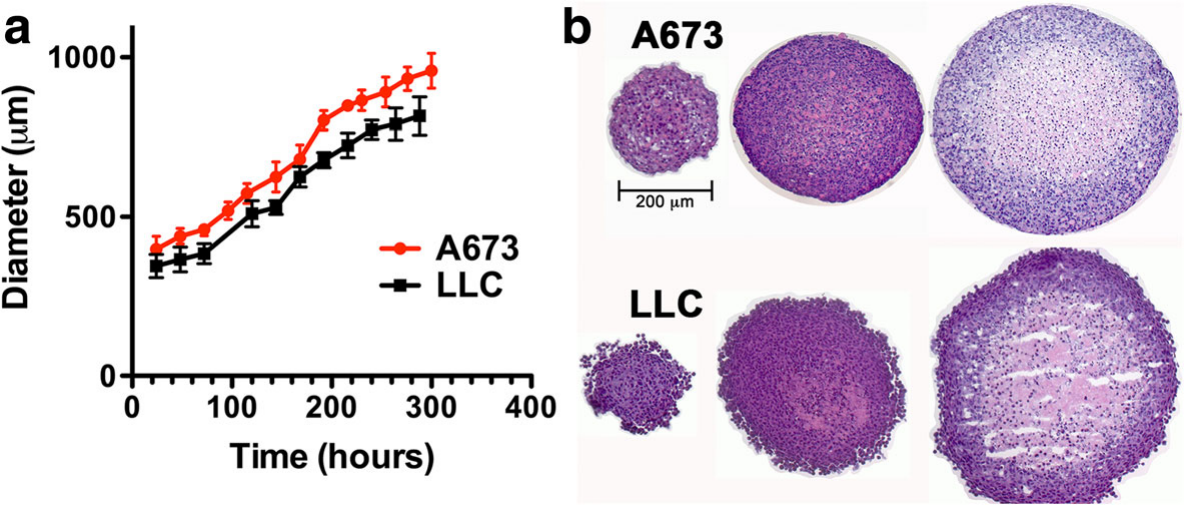
Growth characteristics of A673 and LLC spheroids. **a** Growth curves for spheroids generated with A673 (*red*) and LLC (*black*) cells. The mean diameter and standard deviation from >8 spheroids are plotted over time, from initial formation to final size before spheroid collapse. **b** H&E staining of spheroid cryo-sections reveals the onset of necrosis in spheroids larger than 550 μm. The three sections shown come from spheroids of <500 μm, 550–650 μm and >750 μm in diameter. Shrinkage during processing as well as location of section within spheroids account for the smaller diameter in the final images shown

Growth curves for MCTS were generated using bright-field images captured with a Nikon Eclipse TS100 4× objective (0.1 N.A.). MCTS diameter was determined using ImageJ 1.48v (NIH, Bethesda, MD; http://imagej.nih.gov/ij) and converted to μm. Growth curves in Fig. 1 represent the mean diameter ± standard deviation of at least 8 MCTS from 3 independent experiments.

### Processing of MCTS for histological assessment

MCTS were collected in Eppendorf tubes and allowed to settle at the bottom before culture medium was removed, replaced with freshly thawed 4% paraformaldehyde/phosphate-buffered saline (pH 7.4), and incubated at 4 °C for 3 h. When appropriate, spheroids were incubated with EdU (10 μM, 24 h) or Pimonidazole (100 μM, 3 h) prior to fixation. For cryopreservation, fixed MCTS were dehydrated with 30% sucrose and snap-frozen with Optimal Cutting Temperature media (4583; Tissue Tek, Torrance, CA). Cryosections were made at 5 μm thickness and stored at -80 °C. Morphology of MCTS sections was determined with Harris Hematoxylin and Eosin (PolyScience, Warrington, PA) staining. Glycogen storage determined with Periodic Acid (375,810, Sigma, St. Louis, MO) and Schiff’s Base (3,952,016, Sigma, St. Louis, MO) staining, in parallel to sections pre-incubated with 0.5% amylase. No hematoxylin counter stain was used.

### Immunofluorescence analysis of MCTS sections

Cryo-sections were blocked with antibody specific blocking buffer for 1 h at room temperature in a humidified chamber. Primary antibodies directed against γ-H2AX, Ki-67, cleaved caspase-3, HIF1-α, pATM, and pATR were diluted (1:200) in blocking buffer and incubated on sections for 1 h at room temperature or overnight at 4 °C. After washing with phosphate-buffered saline containing 0.15% Triton X-100, sections were incubated for 1 h at room temperature with fluorescently tagged secondary antibodies diluted (1:200) in blocking buffer, followed by 20-min incubation with Hoechst dye to identify nuclei. Sections were washed and mounted using Fluorgel with DABCO (17985–04; Electron Microscopy Science, Hatfield, PA). Primary antibody dilution for pimonidazole was 1:50. Blocking buffer for γ-H2AX, Ki-67, Hypoxyprobe, and Cleaved Caspase 3 was phosphate-buffered saline supplemented with 10% FBS, 2% BSA, and 0.15% Triton X-100. Blocking for HIF1-α, pATM, pATR was done with phosphate-buffered saline supplemented with 5% donkey serum and 0.15% Triton X-100. EdU was detected following the manufacturer’s instructions (Life Technologies, Carlsbad, CA); MCTS cryo-sections were permeabilized with 0.5% vv^−1^ Triton X-100 followed by incubation with Alexa Fluor 647 azide and Hoechst 33,342 nuclear counter stain.

### ATM and ATR inhibition

10 μM KU55933 or 2.5 μM VE-821 in DMEM supplemented with 2% FBS and containing 0.025% vv^−1^ DMSO were used. Fresh media containing inhibitors was added to MCTS cultures every 48 h. The vehicle control contained 0.025% vv^−1^ DMSO in DMEM supplemented with 2% FBS.

### Western blotting

Cell pellets from exponentially growing cells were lysed by sonication on ice with lysis buffer consisting of 500 mM NaCl, 100 mM HEPES at pH 7.4, 10 mM EDTA, and 0.2% vv^−1^ NP-40, supplemented with phosphatase inhibitors sodium fluoride (NaF; 50 mM) and sodium orthovanadate (1 mM), proteinase inhibitors phenylmethyl sulfonyl fluoride (PMSF, 1 mM), 1× Proteinase Inhibitor Cocktail (P8340; Sigma, St. Louis, MO), and the reducing agent Dithiothreitol (DTT, 1 mM). Cell debris were removed via centrifugation. Protein concentrations were estimated by Bradford assay, samples were electrophoresed on 10% SDS gels and transferred to PVDF membranes. Membranes were blocked using rapid block buffer (M325, Amresco, Solon OH) for 5 min at room temperature followed by incubation with specific primary and secondary antibodies. Western blots were visualized with enhanced chemi-luminescence (34,078; ThermoScientific, Waltham MA) on X-ray films (53PSF; Worldwide Medical, Bristol PA).

### COMET assays

Ten thousand cells suspended in 1% low melting temperature agarose were diluted in TBE (90 mM Tris, 90 mM Boric Acid, 2 mM EDTA, ph 8.5) and spread atop coverslips previously coated in 1% low melting temperature agarose and allowed to solidify for 10 min at 4 °C. Cells were then lysed for 1 h at 4 °C in 2.5 M NaCl, 100 mM EDTA, 10 mM Tris pH 10, 1% wv^−1^ sarkosyl, 1% Triton X-100. Lysed cells were neutralized with TBE. Following alkaline buffer incubation (300 mM NaOH, 1 mM EDTA, pH 12.3) lysates were electropheresed at 25 V for 40 min at room temperature, dehydrated in 70% ethanol and DNA visualized using propidium iodide (1 μg/ml).

### Image acquisition and analysis

Fluorescently stained sections were imaged on a Zeiss confocal microscope at 0.25× magnification across a 1000 μm grid. To determine the number of positive nuclei per spheroid, nuclei were counted as Hoechst-positive cells by using watershed separation and quantification with particle analysis in ImageJ software [12] followed by cell counting to determine the percentage of marker positive nuclei.

### Statistics

Results represent the average of at least 2 separate experiments with a total of at least 8 spheroids per condition ± standard deviation (SD). Statistical analyses were performed using Graphpad PRISM version 5.0 for Mac OSX, http://www.graphpad.com. A t-test was used when two samples/conditions were compared and ANOVA for more than two groups. Significance represents *P* < 0.05.

## Results

### Spheroid growth, the onset of hypoxia and necrosis

Spheroids grown with Lewis lung carcinoma (LLC) and Ewing sarcoma (A673) cell lines were characterized. In each case cells were seeded in appropriate medium (DMEM +2% FBS for A673, and DMEM +10% FBS for LLC). Both A673 and LLC formed loose aggregates within 24 h, and discrete 3D spheroids that could be manipulated within 48 h. Spheroid growth was monitored by light microscopy and plotted in Fig. 1a. A673 cells formed the most tightly packed and well-formed spheroids that plateaued at an average diameter of >950 μm 12 days after seeding. LLC spheroids grew to about 800 μm over 11–12 days, with a less tightly packed surface cell layer that disintegrated as spheroids grew larger.

To monitor the temporal and spatial onset of necrosis and hypoxia, spheroids were harvested and sectioned when they were <500 μm, 500–650 μm and >750 μm in diameter. These time-points were chosen to represent the first formation of a discrete spheroid, an exponential growth phase, and spheroids that had almost reached a size plateau. An initial analysis of cell and spheroid morphology was conducted using haemotoxylin and eosin (H&E) staining (Fig. 1b). A673 and LLC spheroids consisted of uniformly and densely packed cells with onset of necrosis apparent in the center of spheroids that were 550–650 μm in diameter. By the time spheroids were approaching their maximum diameter, large areas of central necrosis were present (accounting for 50–60% of spheroid volume).

**Fig. 2:**
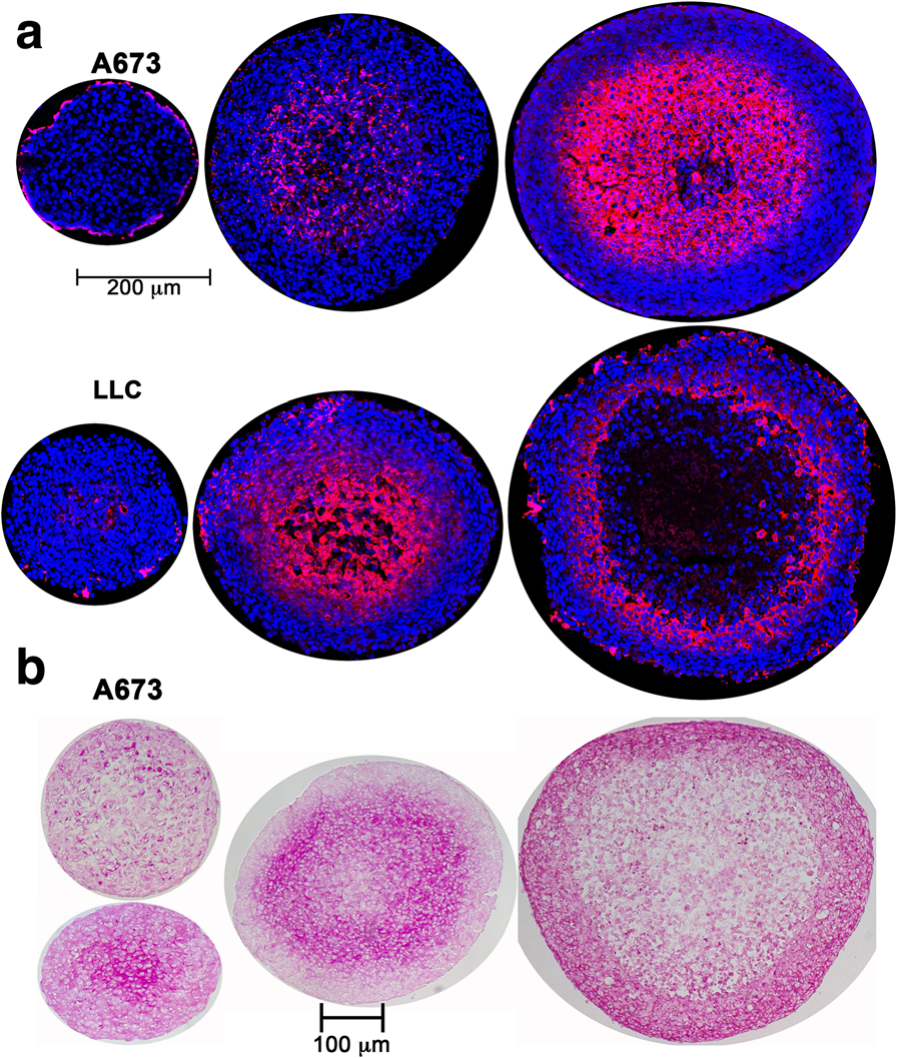
Hypoxia development and glycogen accumulation in growing spheroids. **a** Immunostaining of A673 and LLC spheroid cryo-sections for the hypoxia marker pimonidazole (*red*) shows hypoxia development at different sizes in the central core of A673 and LLC spheroids. Spheroids sections counter-stained with Hoechst 33,342. **b** Periodic Acid Schiff’s Base staining (*dark pink*) demonstrating glycogen storage in MCTS cryo-sections obtained from A673 spheroids of different sizes

Pimonidazole staining was used as a surrogate marker of hypoxia (Fig. 2a). Pimonidazole, a 2-nitroimidazole, is reductively activated in hypoxia and can form stable adducts with proteins, peptides and amino acids. Pimonidazole binding to cellular glutathione occurs at oxygen concentrations below 1.3% [13] and is therefore used as a marker of severe hypoxia. Pimonidazole staining was seen in the core of growing spheroids (>500 μm diameter), and in the necrotic and peri-necrotic regions of the largest spheroids (Fig. 2a). The relationship(s) between spheroid size and the onset of hypoxia and necrosis are similar in both A673 and LLC spheroids. The more detailed analyses described below were restricted to A673, since these cells form more robust and uniform spheroids.

Metabolic adaptations, including glycogen accumulation, are a well-recognized feature of tumors. To examine the spatial relationships between hypoxia in tumor MCTS and glycogen accumulation we evaluated the patterns of periodic acid-Schiff staining in spheroids (Fig. 2b). This is a routine histochemical method for detecting glycogen in tumor samples. In small (~400–500 μm) A673 spheroids PAS staining was observed sporadically throughout the spheroid, with sometimes a greater concentration near the center. In intermediate sized spheroids (550–650 μm) PAS staining became much more restricted to the peri-necrotic region typically denoted as a hypoxic, quiescent zone. In the largest spheroids intense PAS staining was seen at the periphery. Amylase treatment confirmed that PAS staining in these studies corresponded to glycogen accumulation.

### Cell proliferation becomes restricted to the surface as MCTS grow

**Fig. 3:**
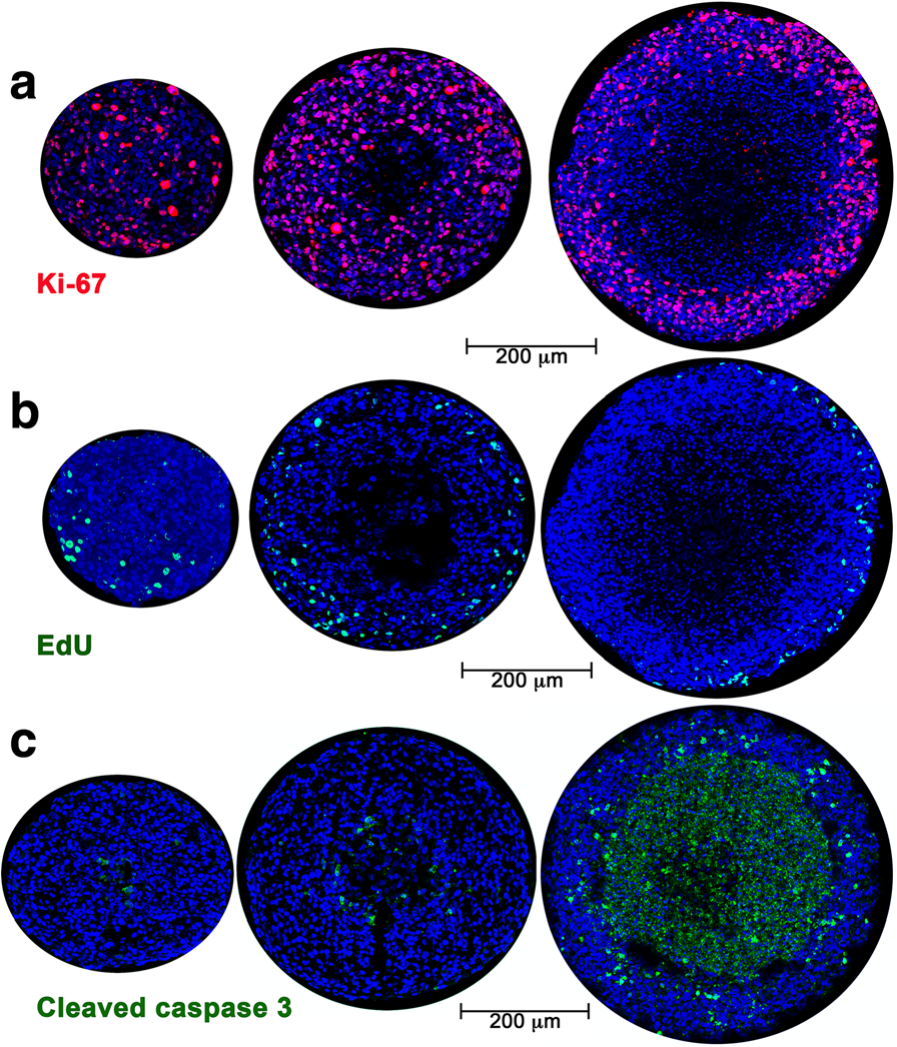
Proliferation and apoptosis in A673 spheroids. **a** Representative images showing the proliferative marker Ki-67 (*red*, with *blue* Hoechst 33,342 counterstain) in A673 spheroids <500 μm, 550–650 μm and >750 μm in diameter. **b** Representative images showing EdU incorporation (*green*, with *blue* Hoechst 33,342 counterstain) in A673 spheroids <500 μm, 550–650 μm and >750 μm in diameter. These are adjacent sections from the same spheroids imaged in Fig. 3a. **c** Cell death in A673 spheroids of <500 μm, 550–650 μm and >750 μm in diameter indicated by staining for cleaved caspase-3 (*green*, with *blue* Hoechst 33,342 counterstain)

Previous studies have noted that the thickness of the viable cell layer in MCTS remains approximately constant as the spheroid grows, while the necrotic core increases proportionally with the size of the spheroid [14]. The viable layer is comprised of an outer proliferative rim that has the best access to nutrients and oxygen, and an inner layer of quiescent non-proliferative but viable cells. A frequently used clinical parameter in tumors is the Ki-67 labeling index (fraction of Ki-67 positive cells). Ki-67 protein is present in all phases of the cell cycle except G0, and is thus a marker for the growth fraction of a cell population. Ki-67 staining was present throughout small (~400 μm) spheroids, but was more restricted to the cells near the spheroid surface once the diameter exceeded 500 μm (Fig. 3a). Ki-67-positive cells were present approximately 150 μm from the spheroid surface in medium sized spheroids, and 70–80 μm in the largest spheroids. This transition appears to coincide with the onset of hypoxia and necrosis in the central core. Interestingly EdU staining, which marks cells in the S-phase, was concentrated in a narrower band along the spheroid surface, in a much more restricted zone than was seen with Ki-67 (Fig. 3b). The failed incorporation of EdU, despite Ki-67 marker expression, indicates a failure to progress through S-phase, perhaps due to checkpoint activation. The apoptosis marker cleaved caspase 3 was used to monitor the onset of cell death in growing spheroids. Paralleling the observations with H&E staining (Fig. 1b), an extensive region of apoptosis was evident in the central core of the largest spheroids (Fig. 3c).

### ATM is primarily responsible for γ-H2AX formation in the hypoxic, peri-necrotic zone of growing spheroids

**Fig. 4:**
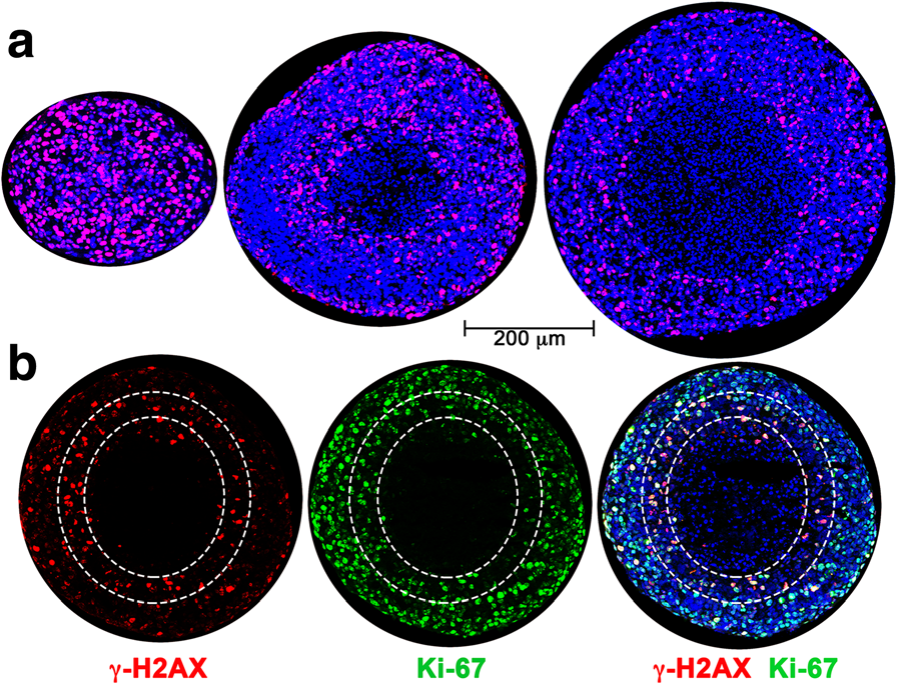
Distribution of γ-H2AX-positive cells in A673 spheroids. **a** γ-H2AX in A673 spheroids was used as a marker of DNA damage repair signaling. Immunostaining of representative spheroids <500 μm, 550–650 μm and >750 μm in diameter is shown. γ-H2AX in red, with blue Hoechst 33,342 counter-stain. **b** γ-H2AX co-staining with Ki-67 in spheroids approximately 570–650 μm in diameter shows activated DDR in proliferative cells within the hypoxic zone. The outer dotted line indicates where Ki-67-positive nuclei decrease in number, inner dotted line marks boundary of viable cells

Nuclear γ-H2AX staining was used to monitor activation of DNA damage repair pathways. γ-H2AX positive nuclei were present throughout the smallest (<500 μm) spheroids (Fig. 4a). It is likely that low levels of replicative stress that occur stochastically during the cell cycle, and is often elevated in tumor cells, could account for this. In larger spheroids two distinct regions of γ-H2AX staining were seen: one prominent band of γ-H2AX positive cells was present in the peri-necrotic hypoxic area, and another smaller population of γ-H2AX positive cells was variably present on the surface of the spheroids (Fig. 4a). Co-staining showed coincidence between most Ki-67-positive and γ-H2AX-positive cells in the peri-necrotic area, indicating that DDR was occurring in proliferating cells (Fig. 4b).

**Fig. 5:**
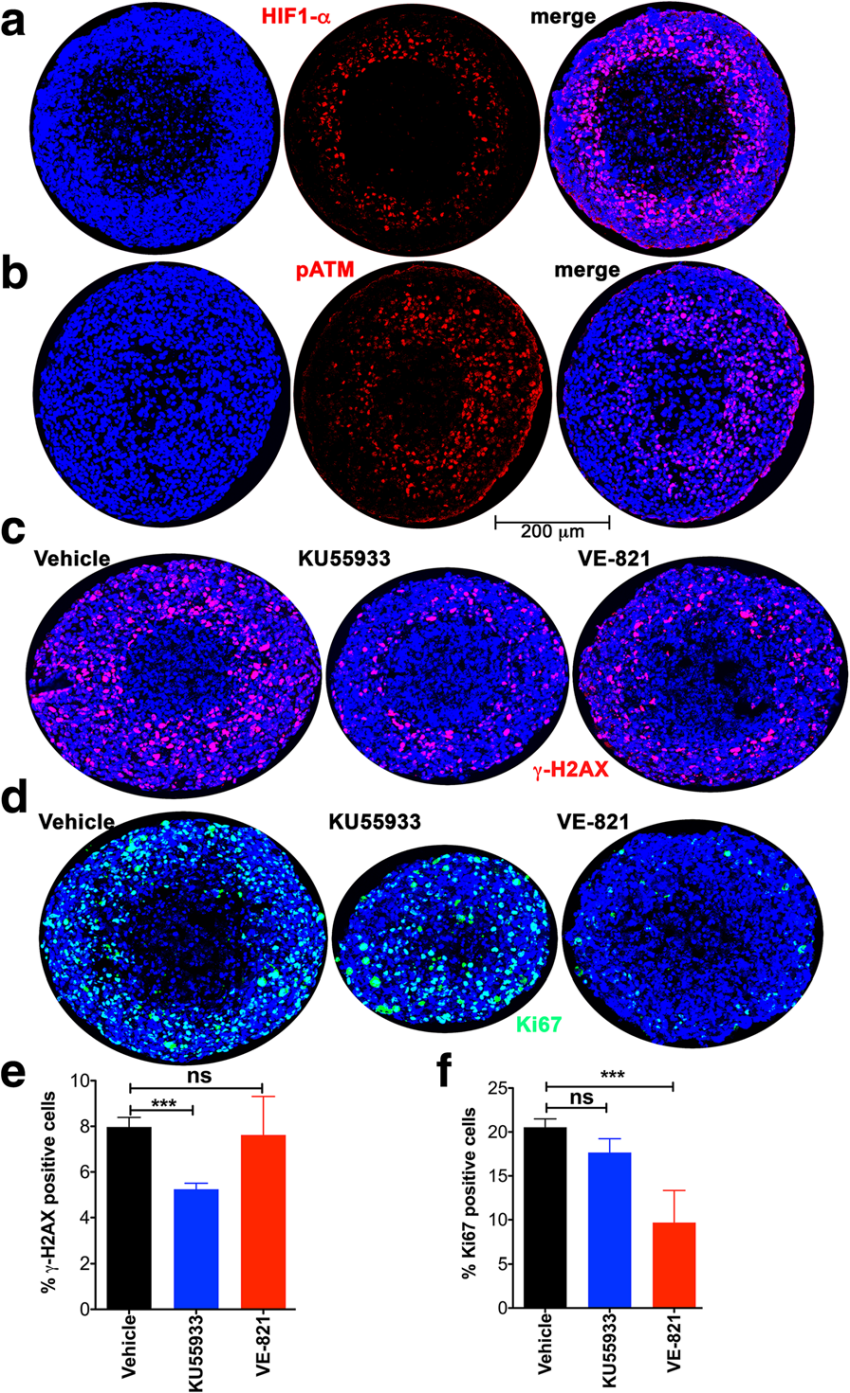
ATM activation is linked to γ-H2AX formation while ATR activity contributes to proliferation in A673 spheroids. **a** Activation of the hypoxia-responsive factor HIF1-α (*red* with *blue* Hoechst 33,342 counter-stain) is observed in the peri-necrotic zone of A673 spheroids 570–650 μm in diameter. **b** Activation of ATM (phosphorylation at serine 1981 (pATM); *red* with *blue* Hoechst 33,342 counter-stain) is seen in the hypoxic, peri-necrotic zone. **c** The distribution of γ-H2AX (*red*, with *blue* Hoechst 33,342 counter-stain) in spheroids maintained for 96 h in either vehicle, the ATM inhibitor KU55933, or the ATR inhibitor VE-821. Spheroids were <500 μm in diameter at the start of the experiment and vehicle-treated spheroids were approximately 700 μm in diameter at the end of 96 h. Both KU55933 and VE-821 impaired spheroid growth. **d** The distribution of Ki-67 (*green*, with *blue* Hoechst 33,342 counter-stain) in spheroids maintained for 96 h in either vehicle, the ATM inhibitor KU55933, or the ATR inhibitor VE-821. **e** Bar graph showing percentage of γ-H2AX-positive cells per spheroid when maintained for 96 h in either vehicle, the ATM inhibitor KU55933, or the ATR inhibitor VE-821. Mean and standard deviation are shown; each bar represents at least 15 spheroids from three independent experiments. **f** Percentage of Ki-67-positive cells per spheroid when maintained for 96 h in either vehicle, the ATM inhibitor KU55933, or the ATR inhibitor VE-821 is shown as the mean and standard deviation; each bar represents at least 15 spheroids from three independent experiments. One way ANOVA with Dunnett’s post-test. ***P < .001, ns *P* > .05

Based on previous studies in 2D culture systems [2, 4, 5, 15–18] it is reasonable to hypothesize that the γ-H2AX staining in the interior of spheroids is a result of the hypoxia-induced activation of the ATR/ATM kinases. Growing spheroids (500–650 μm diameter) were stained with antibodies towards the hypoxia marker HIF1-α, pATM (phospho-serine 1981), pATR (phospho-serine 428), and pChk1 (phospho-serine 345). The hypoxic, HIF1-α-positive region (Fig. 5a) coincided with extensive phosphorylation of ATM (Fig. 5b) in the peri-necrotic zone. We were unable to detect either pATR (pSer428) or pChk1 (pSer345) in spheroids. This observation is consistent with compelling evidence that phosphorylation at ATR Ser-428 is not associated with activation of its kinase activity [19, 20], although Ser-428 phosphorylation has been associated with DNA damage. To further evaluate the roles of ATM and ATR in γ-H2AX formation we used pharmacological inhibitors; KU55933 specific to ATM [21] and VE-821 specific to ATR [22]. Pre-formed spheroids (<450 μm) were chosen for the start of inhibitor treatment as it represented a time-point just before the development of hypoxia or significant necrosis. Spheroids were maintained in either vehicle or ATM/ATR inhibitor. Both KU55933 and VE-821 treatment inhibited the growth of spheroids. After 5 days of treatment with either KU55933 or VE-821 spheroids stopped growing any further (reaching a plateau at an average diameter of 660 μm), while vehicle-treated spheroids typically increased in diameter by about 25% over 5 more days. Spheroids were stained for γ-H2AX after 96 h of inhibitor treatment. Spheroids treated with the ATM inhibitor had fewer γ-H2AX positive cells, but the spatial distribution did not change (Fig. 5c, e). VE-821 (ATR inhibition) had negligible effect on the percentage of γ-H2AX positive cells (Fig. 5c, e). Interestingly, while KU55933 had no significant effect on the percentage of Ki-67-positive cells in the spheroids, VE-821 decreased it by over 50% suggesting a strong effect on the proliferative index (Fig. 5d, f). Together these observations support a role for ATM in H2AX phosphorylation in the hypoxic zone of spheroids, while ATR plays a role in proliferation.

### Hypoxia-induced γ-H2AX formation is differentially affected by ATM and ATR inhibition in spheroids versus two-dimensional cultures

**Fig. 6:**
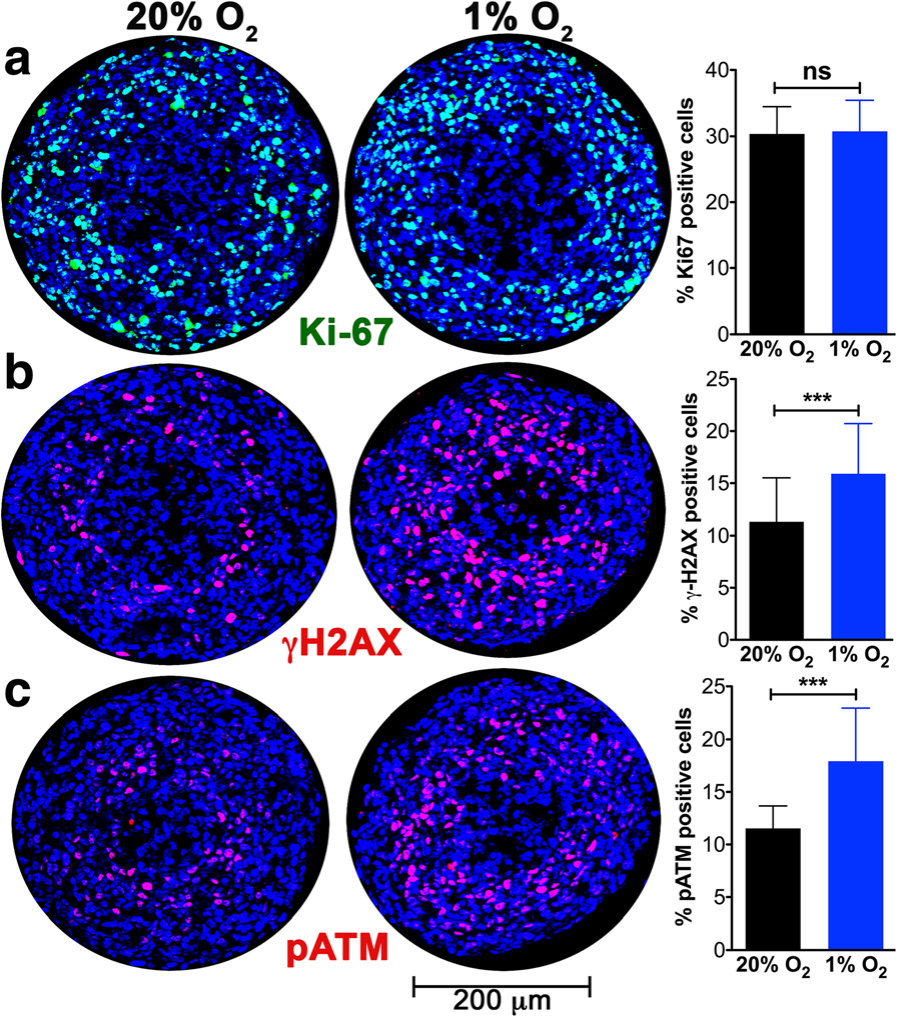
ATM activation and γ-H2AX formation are promoted by hypoxia, while proliferation is unaffected. **a** The effect of maintaining ~400 μm A673 spheroids in 1% O_2_ for 12 h was monitored. Spheroid sections were stained for the proliferation marker Ki-67 (*green*). The % Ki-67-positive cells is plotted (mean and standard deviation from >12 spheroids and two independent experiments). Hypoxia had no effect on cell proliferation. **b** The DDR marker γ-H2AX (*red*) is upregulated in the core of spheroids maintained in 1% O_2_ for 12 h. Bar graph shows the mean and standard deviation from >45 spheroids and >5 independent experiments. **c** Activation of ATM kinase is indicated by staining for pATM (*red*) in spheroids maintained in 1% O_2_ for 12 h. Bar graph shows the mean and standard deviation from >15 spheroids and 4 independent experiments. One way ANOVA with Dunnett’s post-test. ***P < .001, ns *P* > .05

To specifically query whether the changes in distribution of DDR markers with increasing spheroid size were a direct result of hypoxia at the core of the spheroid, we examined the effect of incubating small (<500 μm) spheroids (before the onset of hypoxia or necrosis) in 1% O_2_ for 12 h. They were then sectioned and probed for γ-H2AX and Ki-67. Interestingly, the percentage or distribution of cells staining positive for Ki-67 did not change significantly when maintained in 1% O_2_ (Fig. 6a). Spheroids subject to hypoxia had significantly more γ-H2AX staining in the central core while there was no change in γ-H2AX staining on the surface (Fig. 6b). Oxygen diffusion into a spheroid is limited by inter-cellular tight junctions and senescent/necrotic cells. Direct measurements of oxygen levels within spheroids have shown that as spheroid size increases there is a gradient of oxygen tension from the surface to the center [23]. Hence it is likely that, while 1% O_2_ is not sufficient to trigger phosphorylation of H2AX, the core of the spheroid has reached a threshold level of O_2_ required for γ-H2AX formation. A similar trend was seen for the activation of ATM kinase; there was a significant increase in pATM staining in the core of spheroids subject to hypoxia (Fig. 6c).

**Fig. 7:**
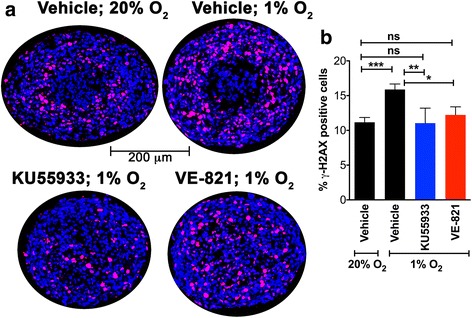
ATM or ATR inhibition attenuates the severe hypoxia-induced increase in γ-H2AX levels in spheroids. **a** A673 spheroids ~400 μm in diameter were maintained for 12 h in either 20% O_2_ or 1% O_2_ in vehicle, 1% O2 in KU55933 or 1% O2 in VE-821. The effect of ATM inhibition (KU55933) or ATR inhibition (VE-821) on γ-H2AX distribution (*red*, with *blue* Hoechst 33,342 counter-stain) is shown. **b** Percentage of γ-H2AX-positive cells in spheroids was quantitated. Bar graph shows the mean and standard deviation from >14 spheroids and at least 3 independent experiments. One way ANOVA with Dunnett’s post-test. ***P < .001, **P < .01, ns *P* > .05

To further probe the role of ATM kinase activity in hypoxia-induced γ-H2AX formation spheroids were treated with the ATM inhibitor KU55933 during the 12 h in which they were maintained in 1% O_2_ (Fig. 7a). Control spheroids maintained in vehicle for the 12 h of hypoxia displayed an increase in γ-H2AX-positive cells, while there was no increase in γ-H2AX when the spheroids were subject to 1% O_2_ in the presence of KU55933 (Fig. 7b). These observations, along with the results presented in Fig. 5c are consistent with hypoxia-induced activation of ATM contributing to the H2AX Ser-139 phosphorylation in the spheroid center. The ATR inhibitor VE-821 also inhibited hypoxia-induced γ-H2AX formation in spheroids maintained in 1% O_2_ (Fig. 7b). This contrasts with the results seen with VE-821 in Fig. 5c, raising the possibility that ATR plays a role in γ-H2AX formation only under conditions not attained when spheroids are maintained at 20% O_2_.

**Fig. 8:**
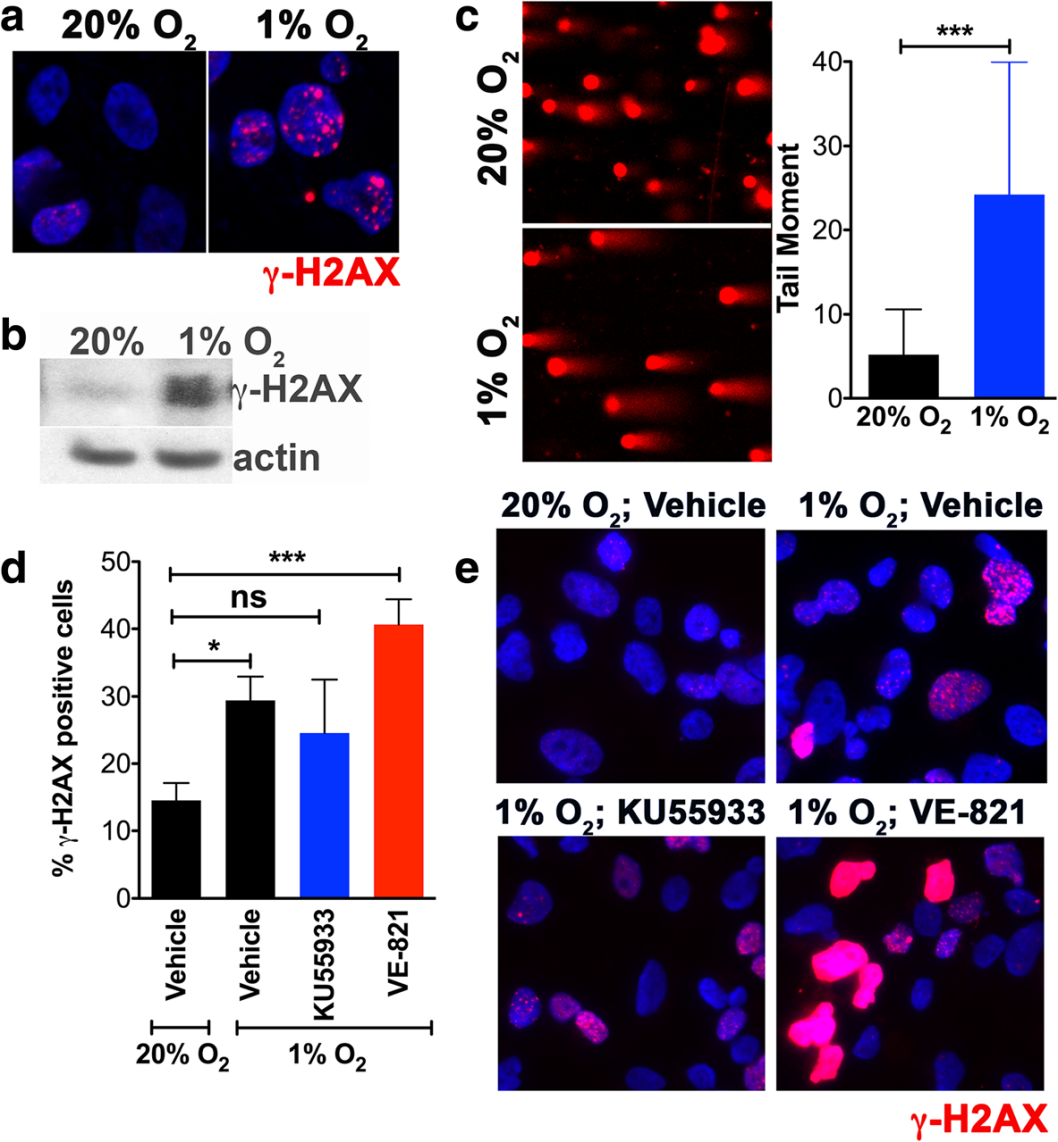
1% O_2_ is sufficient to induce DNA damage in A673 monolayers and inhibition of ATR promotes H2AX phosphorylation. **a** The effect of maintaining monolayers of A673 cells in 1% O_2_ for 12 h was monitored. Immunofluorescence indicates the formation of distinct γ-H2AX foci (*red*) in A673 cells. **b** Western blot shows elevation of γ-H2AX levels in A673 cells maintained in 1% O_2_ for 12 h. **c** Alkaline COMET assay shows induction of COMET tails (a direct measure of DNA damage) when A673 cells were maintained in 1% O_2_ for 12 h. Tail moments were quantitated using OpenCOMET [43] and are plotted as the mean and standard deviation. **d** Bar graph showing the percentage of γ-H2AX-positive A673 cells when monolayers were maintained for 12 h in either 20% O_2_ or 1% O_2_ in vehicle, 1% O_2_ in KU55933 (ATM inhibitor), or 1% O2 in VE-821 (ATR inhibitor). Mean and standard deviation from two independent experiments are shown. **e** Immunofluorescence for γ-H2AX (*red*) in A673 monolayers maintained for 12 h in either 20% O_2_ or 1% O_2_ in vehicle, 1% O2 in KU55933 (ATM inhibitor), or 1% O2 in VE-821 (ATR inhibitor). ATR inhibition under hypoxia results in cells with either intense pan-nuclear γ-H2AX or distinct foci, while only distinct γ-H2AX foci are seen under hypoxia in the presence of either vehicle or ATM inhibitor. One way ANOVA with Tukey’s multi-comparison post-test. ***P < .001, **P < .01, *P < .05, ns *P* > .05

Previous studies have shown that moderate hypoxia (defined as >0.1% O2), such as that used here, can lead to activation of ATR and ATM kinases, without inducing COMET assay-detectable DNA damage in monolayer cultures [2, 16]. In these instances, γ-H2AX is not localized in foci consistent with the absence of nucleating DNA damage. Sections from spheroids do not provide the resolution necessary to clearly identify γ-H2AX foci, if they are present. We examined how A673 cells in 2D culture respond to hypoxia (1% O_2_ for 12 h). Western blots showed hypoxia-induced γ-H2AX formation (Fig. 8b), and examination of cells by immunofluorescence revealed that γ-H2AX foci are formed (Fig. 8a). Further, alkaline COMET assays showed that A673 cells maintained in 1% O_2_ for 12 h had significantly increased tail moments consistent with the induction of DNA damage (Fig. 8c). In 2D culture, treatment of A673 cells with KU55933 attenuated the hypoxia-induced increase in the percentage of γ-H2AX-positive cells (Fig. 8d, e). In contrast, presence of the ATR inhibitor, VE-821, led to much higher levels of γ-H2AX induction under hypoxia, characterized by cells with both distinct foci and pan-nuclear γ-H2AX staining (Fig. 8d, e).

## Discussion

Solid tumors are characterized by the presence of both oxic and hypoxic regions. The hypoxic tumor microenvironment stimulates neo-angiogenesis, and tumor growth becomes linked to the availability of vasculature-supplied oxygen and nutrients. For this reason anti-angiogenics have been a major focus of cancer drug development efforts. Recent evidence also links hypoxia, DNA damage repair and pathological angiogenesis in both tumors and proliferative retinopathies [10, 24, 25]. While there have been some spectacular clinical successes with anti-angiogenics in cancer treatment, the overall and progression-free survival rates have been disappointing [26]. Among the factors contributing to this are adaptations of cancer cells in the hypoxic tumor microenvironment that allow them to proliferate and to become more invasive and resistant to chemo- and radio-therapy. Multiple studies have tried to correlate tumor tissue pO_2_, distance from blood vessels, and proliferation indices in attempts to derive a correlation between oxygen tension and proliferative or quiescent state of tumor cells. No clear consensus has emerged from these studies [27]. Multicellular tumor spheroids offer a convenient system in which to model such hypoxic tumor niches in vitro. Here we sought to examine markers of DNA damage in spheroids and their spatial correlation with hypoxia, glycogen accumulation, proliferation, necrosis and apoptosis.

Typically, two distinct zones are formed as spheroids grow: a viable ring of cells on the surface and a central necrotic core. The H&E staining pattern and the distribution of Ki-67 staining described here is consistent with this model and with previous studies using MCTS to examine the regionalization of cell proliferation [28, 29]. We also see incorporation of EdU in a much more restricted ring of cells on the spheroid surface than that seen with Ki-67 staining (Fig. 3), as previously reported in spheroids formed with HCT116 (colon adenocarcinoma) cells [29] and Capan-2 (pancreatic cancer) cells [28]. Experimental measurements and mathematical modeling have shown that spheroids larger than about 200 μm have steep oxygen gradients; the center of the spheroid is anoxic and the interface of the hypoxic and proliferating zones is mildly hypoxic (oxygen partial pressure is predicted to be about 10 mmHg or 1.3% O_2_) [30]. The presence of tumor cells with proliferative capacity under such intermediate hypoxia has been associated with tumor aggressiveness and lower disease-free survival rates [31]. The 3D culture system used here identifies such a proliferative cell population in the peri-necrotic, hypoxic region of growing spheroids (Fig. 4). Our data show that these cells also stain positive for γ-H2AX (Ser-139 phosphorylated H2AX) indicating the assembly of DDR complexes. γ-H2AX has been used extensively as a marker of DNA double strand breaks due to its early formation at sites of DNA lesions following irradiation or other means of double strand break induction. H2AX Ser-139 phosphorylation allows it to recruit DNA damage repair proteins, facilitating repair and survival of cells (reviewed in [32]). The coincidence of DDR markers with proliferation in spheroids is indicative of a productive DDR process that permits survival of some hypoxic cells. The peri-necrotic, proliferating cells (Fig. 4) are also within a ring of HIF1-α -positive cells (Fig. 5a), confirming the hypoxic micro-environment and identifying the region within a spheroid where transcriptional programs regulated by HIF1-α are likely to be upregulated. HIF1-α is known to promote glycogen accumulation in both tumor and non-tumor cells [33–35]. Furthermore, treatment of xenografts with the anti-angiogenic agent Bevacizumab led not only to increased hypoxia, but also to an increase in glycogen and upregulation of genes involved in glycogen metabolism. Glycogen is linked to increased proliferation and to survival under conditions of hypoxia and glucose deprivation [34, 35]. In agreement with these reports, we noted glycogen accumulation in the peri-necrotic region of growing spheroids (Fig. 2b) coincident with the ring of HIF1-α staining seen in Fig. 5a. Curiously in the largest spheroids examined, PAS-staining (hence glycogen accumulation) was present in the entire viable zone, implying a broader cellular adaptation to use glycogen as an energy store. These observations regarding the regionalization of glycogen accumulation within a tumor spheroid are of particular relevance given the interest in targeting glycogen metabolism for cancer treatment [36]. Also pertinent is the fact that the cells used in this study derive from a human Ewing sarcoma, a malignancy for which cytoplasmic glycogen accumulation (as detected by PAS-staining) is a diagnostic parameter.

The (phosphatidylinositol 3-kinase)-like (PI3KK; includes ATM, ATR and DNA-PK) family of kinases are responsible for H2AX-Ser-139 phosphorylation, a critical early step in the DDR pathway. While there is crosstalk between these kinases, they are each associated with distinct stress conditions. ATM typically phosphorylates H2AX in response to double-strand breaks while ATR is activated in replication stress and the presence of single-stranded DNA. DNA-PK is most commonly associated with hypertonic conditions and apoptotic fragmentation. Both ATM and ATR are known to be activated by severe (O_2_ < 0.1%) hypoxia [2, 5, 15, 16, 18]. In the case of ATR, this is likely to be a result of replication stress leading to regions of single-strand DNA. The exact mechanism by which ATM is activated under hypoxia is not known, although some evidence suggests that it is a response to the combined presence of replication stress and heterochromatin modifications stimulated by hypoxia [9]. The distribution of activated ATM shown here in growing spheroids with a hypoxic and necrotic center is informative. pATM is concentrated in the peri-necrotic, hypoxic area (Fig. 5b). ATM is known to phosphorylate and stabilize HIF1-α under hypoxic conditions [8] and this pATM-positive region is also populated by HIF1-α-positive cells. We were unable to detect activated ATR (pATR) in growing spheroids, despite it’s known role in unstressed replication [18, 37]. Consistent with these observations pharmacological inhibition of ATM reduced γ-H2AX levels, whereas ATR inhibition altered cell proliferation (Ki-67 staining) rather than the levels or distribution of H2AX-positive cells (Fig. 5). In 2D culture, ATR inhibition under hypoxic conditions led to increased γ-H2AX as well as a significant number of cells showing pan-nuclear γ-H2AX staining. UV-irradiation has previously been shown to induce similar pan-nuclear γ-H2AX staining that represented a pre-apoptotic state [38]. As with results reported here, ATR inhibition increased UV-induced pan-nuclear γ-H2AX. The ATR inhibitor VE-821 is also known to decrease the percentage of cells in the S-phase of the cell cycle under normal conditions, and to abrogate the DNA damage-induced checkpoint (G2/M) after irradiation and hypoxia [22, 39], thus reducing cell survival. This effect is particularly pronounced in p53-deficient cells; the Ewing Sarcoma cell line A673 used herein, contains both the oncogenic transcription factor EWS/FlI1 and mutations in the TP53 gene making them p53-null [40]. This combination of increased replication and impaired G1 checkpoint signaling may explain why hypoxic stress resulted in observable DNA damage. Further, the EWS/FLI1 transcription factor induces replication stress and the formation of stalled replication forks. Hence Ewing sarcoma cells are highly sensitive to ATR inhibitors [41].

Hypoxic activation of ATR also suppresses DNA replication through a cdc25a-CDK2-APC/C^Cdh1^-cdc6 mechanism [42]. Both ATR and ATM inhibition retarded spheroid growth in our studies; a similar effect on spheroid growth has previously been reported using glioblastoma cells [37]. Taken together these results indicate that ATM activity contributes to hypoxia-induced γ-H2AX formation in the MCTS core and that ATR activity is important for cell proliferation. The observation that ATR and ATM inhibitors have different effects on DDR in monolayer cells compared to MCTS highlights the importance of recreating a 3D tumor environment when validating therapeutic targets.

## Conclusions

MCTS can be used to mimic tumor microenvironments such as the anoxic, hypoxic and oxic niches within solid tumors, as well as populations of cells that are viable and proliferating under these different oxygen tensions. Using this in vitro model system we show that ATM, but not ATR, is the primary kinase responsible for γ-H2AX formation in the hypoxic core of A673 MCTS, and that targeting either ATM or ATR kinase inhibits spheroid growth, albeit through different mechanisms. The results obtained using MCTS are distinct and complimentary to those seen with 2D culture. MCTS offer unique advantages in testing therapeutics designed to target malignant cells that evade conventional treatment strategies by adapting to the hypoxic tumor microenvironment.

## Abbreviations

APC/C: Anaphase-Promoting Complex
ATM: Ataxia Telangiectasia Mutated
ATR: ATM and Rad3-related
Cdc25a: Cell division cycle 25 homolog A
Cdc6: Cell Division Cycle 6
Cdh1: APC/C activation protein
CDK2: Cyclin-dependent kinase 2
Chk1: Checkpoint Kinase 1
Chk2: Checkpoint Kinase 2
DDR: DNA damage repair
DMEM: Dulbecco’s Modified Eagles Medium
DNA-PK: DNA-dependent protein kinase
EdU: 5-Ethynyl-2′-deoxyuridine
EWS/FLi1: Fusion protein of the Ewing sarcoma breakpoint region 1 gene product and the Friend of Leukemia 1 protein
H&E: Hematoxylin and Eosin Staining
HIF1-α: Hypoxia Inducible Factor 1α
LLC: Lewis Lung Carcinoma cells
MCTS: Multi-Cellular Tumor Spheroids
PAS: Periodic-acid and Schiff’s Base staining
pATM: Phosphorylated ATM Serine-1981
pATR: Phosphorylated ATR Serine-428
pChk1: Phosphorylated Chk1 Serine-345
pO_2_: Oxygen partial pressure
ROS: Reactive Oxygen Species
VEGF: Vascular Endothelial Growth Factor
γ-H2AX: Phosphorylated H2AX-Serine-139

## References

[1] Brown JM (2007). Tumor hypoxia in cancer therapy. Methods Enzymol.

[2] Hammond EM, Dorie MJ, Giaccia AJ (2003). ATR/ATM targets are phosphorylated by ATR in response to hypoxia and ATM in response to reoxygenation. J Biol Chem.

[3] Scanlon SE, Glazer PM (2015). Multifaceted control of DNA repair pathways by the hypoxic tumor microenvironment. DNA Repair (Amst).

[4] Olcina M, Lecane PS, Hammond EM (2010). Targeting hypoxic cells through the DNA damage response. Clinical cancer research : an official journal of the American Association for Cancer Research.

[5] Olcina MM, Grand RJ, Hammond EM (2014). ATM activation in hypoxia - causes and consequences. Mol Cell Oncol.

[6] Bindra RS, Crosby ME, Glazer PM (2007). Regulation of DNA repair in hypoxic cancer cells. Cancer Metastasis Rev.

[7] Hammond EM, Giaccia AJ (2004). The role of ATM and ATR in the cellular response to hypoxia and re-oxygenation. DNA Repair (Amst).

[8] Cam H, Easton JB, High A, Houghton PJ (2010). mTORC1 signaling under hypoxic conditions is controlled by ATM-dependent phosphorylation of HIF-1alpha. Mol Cell.

[9] Olcina MM, Foskolou IP, Anbalagan S, Senra JM, Pires IM, Jiang Y, Ryan AJ, Hammond EM (2013). Replication stress and chromatin context link ATM activation to a role in DNA replication. Mol Cell.

[10] Economopoulou M, Langer HF, Celeste A, Orlova VV, Choi EY, Ma M, Vassilopoulos A, Callen E, Deng C, Bassing CH (2009). Histone H2AX is integral to hypoxia-driven neovascularization. Nat Med.

[11] LaBarbera DV, Reid BG, Yoo BH (2012). The multicellular tumor spheroid model for high-throughput cancer drug discovery. Expert Opin Drug Discov.

[12] Schneider CA, Rasband WS, Eliceiri KW (2012). NIH image to ImageJ: 25 years of image analysis. Nat Methods.

[13] Gross MW, Karbach U, Groebe K, Franko AJ, Mueller-Klieser W (1995). Calibration of misonidazole labeling by simultaneous measurement of oxygen tension and labeling density in multicellular spheroids. Int J Cancer.

[14] Conger AD, Ziskin MC (1983). Growth of mammalian multicellular tumor spheroids. Cancer Res.

[15] Bencokova Z, Kaufmann MR, Pires IM, Lecane PS, Giaccia AJ, Hammond EM (2009). ATM activation and signaling under hypoxic conditions. Mol Cell Biol.

[16] Hammond EM, Denko NC, Dorie MJ, Abraham RT, Giaccia AJ (2002). Hypoxia links ATR and p53 through replication arrest. Mol Cell Biol.

[17] Hammond EM, Green SL, Giaccia AJ (2003). Comparison of hypoxia-induced replication arrest with hydroxyurea and aphidicolin-induced arrest. Mutat Res.

[18] Pires IM, Bencokova Z, Milani M, Folkes LK, Li JL, Stratford MR, Harris AL, Hammond EM (2010). Effects of acute versus chronic hypoxia on DNA damage responses and genomic instability. Cancer Res.

[19] Liu S, Shiotani B, Lahiri M, Marechal A, Tse A, Leung CC, Glover JN, Yang XH, Zou L (2011). ATR autophosphorylation as a molecular switch for checkpoint activation. Mol Cell.

[20] Nam EA, Zhao R, Glick GG, Bansbach CE, Friedman DB, Cortez D (2011). Thr-1989 phosphorylation is a marker of active ataxia telangiectasia-mutated and Rad3-related (ATR) kinase. J Biol Chem.

[21] Hickson I, Zhao Y, Richardson CJ, Green SJ, Martin NM, Orr AI, Reaper PM, Jackson SP, Curtin NJ, Smith GC (2004). Identification and characterization of a novel and specific inhibitor of the ataxia-telangiectasia mutated kinase ATM. Cancer Res.

[22] Reaper PM, Griffiths MR, Long JM, Charrier JD, Maccormick S, Charlton PA, Golec JM, Pollard JR (2011). Selective killing of ATM- or p53-deficient cancer cells through inhibition of ATR. Nat Chem Biol.

[23] Langan LM, Dodd NJ, Owen SF, Purcell WM, Jackson SK, Jha AN (2016). Direct measurements of oxygen gradients in spheroid culture system using electron parametric resonance oximetry. PLoS One.

[24] Wang Y, Tadjuidje E, Pandey RN, Stefater JA 3rd, Smith LE, Lang RA, Hegde RS. The Eyes Absent Proteins in Developmental and Pathological Angiogenesis. The American journal of pathology. 2016; 10.1016/j.ajpath.2015.10.031 PMC4816707 26765957

[25] Rankin EB, Giaccia AJ, Hammond EM (2009). Bringing H2AX into the angiogenesis family. Cancer Cell.

[26] Bergers G, Hanahan D (2008). Modes of resistance to anti-angiogenic therapy. Nat Rev Cancer.

[27] Evans SM, Jenkins KW, Chen HI, Jenkins WT, Judy KD, Hwang WT, Lustig RA, Judkins AR, Grady MS, Hahn SM (2010). The relationship among hypoxia, proliferation, and outcome in patients with de novo glioblastoma: a pilot study. Transl Oncol.

[28] Laurent J, Frongia C, Cazales M, Mondesert O, Ducommun B, Lobjois V (2013). Multicellular tumor spheroid models to explore cell cycle checkpoints in 3D. BMC Cancer.

[29] Gomes A, Guillaume L, Grimes DR, Fehrenbach J, Lobjois V, Ducommun B (2016). Oxygen partial pressure is a rate-limiting parameter for cell proliferation in 3D spheroids grown in Physioxic culture condition. PLoS One.

[30] Grimes DR, Kelly C, Bloch K, Partridge M (2014). A method for estimating the oxygen consumption rate in multicellular tumour spheroids. J R Soc Interface.

[31] Hoogsteen IJ, Marres HA, Wijffels KI, Rijken PF, Peters JP, van den Hoogen FJ, Oosterwijk E, van der Kogel AJ, Kaanders JH (2005). Colocalization of carbonic anhydrase 9 expression and cell proliferation in human head and neck squamous cell carcinoma. Clin Cancer Res.

[32] Scully R, Xie A (2013). Double strand break repair functions of histone H2AX. Mutat Res.

[33] Shen GM, Zhang FL, Liu XL, Zhang JW (2010). Hypoxia-inducible factor 1-mediated regulation of PPP1R3C promotes glycogen accumulation in human MCF-7 cells under hypoxia. FEBS Lett.

[34] Pelletier J, Bellot G, Gounon P, Lacas-Gervais S, Pouyssegur J, Mazure NM (2012). Glycogen synthesis is induced in hypoxia by the hypoxia-inducible factor and promotes cancer cell survival. Front Oncol.

[35] Pescador N, Villar D, Cifuentes D, Garcia-Rocha M, Ortiz-Barahona A, Vazquez S, Ordonez A, Cuevas Y, Saez-Morales D, Garcia-Bermejo ML (2010). Hypoxia promotes glycogen accumulation through hypoxia inducible factor (HIF)-mediated induction of glycogen synthase 1. PLoS One.

[36] Zois CE, Harris AL (2016). Glycogen metabolism has a key role in the cancer microenvironment and provides new targets for cancer therapy. J Mol Med (Berl).

[37] Pires IM, Olcina MM, Anbalagan S, Pollard JR, Reaper PM, Charlton PA, McKenna WG, Hammond EM (2012). Targeting radiation-resistant hypoxic tumour cells through ATR inhibition. Br J Cancer.

[38] de Feraudy S, Revet I, Bezrookove V, Feeney L, Cleaver JE (2010). A minority of foci or pan-nuclear apoptotic staining of gammaH2AX in the S phase after UV damage contain DNA double-strand breaks. Proc Natl Acad Sci U S A.

[39] Prevo R, Fokas E, Reaper PM, Charlton PA, Pollard JR, McKenna WG, Muschel RJ, Brunner TB (2012). The novel ATR inhibitor VE-821 increases sensitivity of pancreatic cancer cells to radiation and chemotherapy. Cancer Biol Ther.

[40] May WA, Grigoryan RS, Keshelava N, Cabral DJ, Christensen LL, Jenabi J, Ji L, Triche TJ, Lawlor ER, Reynolds CP (2013). Characterization and drug resistance patterns of Ewing's sarcoma family tumor cell lines. PLoS One.

[41] Nieto-Soler M, Morgado-Palacin I, Lafarga V, Lecona E, Murga M, Callen E, Azorin D, Alonso J, Lopez-Contreras AJ, Nussenzweig A (2016). Efficacy of ATR inhibitors as single agents in Ewing sarcoma. Oncotarget.

[42] Martin L, Rainey M, Santocanale C, Gardner LB (2012). Hypoxic activation of ATR and the suppression of the initiation of DNA replication through cdc6 degradation. Oncogene.

[43] Gyori BM, Venkatachalam G, Thiagarajan PS, Hsu D, Clement MV (2014). OpenComet: an automated tool for comet assay image analysis. Redox Biol.

